# The Biodiversity of Grapevine Bacterial Endophytes of *Vitis amurensis* Rupr.

**DOI:** 10.3390/plants11091128

**Published:** 2022-04-21

**Authors:** Olga A. Aleynova, Nikolay N. Nityagovsky, Alexandra S. Dubrovina, Konstantin V. Kiselev

**Affiliations:** Laboratory of Biotechnology, Federal Scientific Center of the East Asia Terrestrial Biodiversity, Far Eastern Branch of the Russian Academy of Sciences, Vladivostok 690022, Russia; niknit1996@gmail.com (N.N.N.); dubrovina@biosoil.ru (A.S.D.); kiselev@biosoil.ru (K.V.K.)

**Keywords:** bacteria, endophytes, grape, microbiome, *Vitis amurensis*

## Abstract

In this paper, the composition profiles of bacterial endophytes in wild-growing Amur grape *Vitis amurensis* Rupr. grown in the south of the Russian Far East were analyzed using both a cultivation-dependent (sowing bacteria) and a cultivation-independent (next generation sequencing, NGS) approach. Both methods revealed the prevalent endophytes in *V. amurensis* were represented by *Gammaproteobacteria*—40.3–75.8%, *Alphaproteobacteria*—8.6–18.7%, *Actinobacteria*—9.2–15.4%, and *Bacilli*—6.1–6.6%. NGS also showed a large proportion of *Bacteroidia* (12.2%) and a small proportion of other classes (less than 5.7%). In general, NGS revealed a greater variety of classes and genera in the endophytic bacterial community due to a high number of reads (574,207) in comparison with the number of colonies (933) obtained after the cultivation-dependent method. A comparative analysis performed in this study showed that both wild grape *V. amurensis* from Russia and domesticated cultivars of *V. vinifera* from Germany and California (USA) exhibit the same basic composition of endophytic bacteria, while the percentages of major taxa and minor taxa showed some differences depending on the plant organ, grape individuals, environmental conditions, and sampling time. Furthermore, the obtained data revealed that lower temperatures and increased precipitation favored the number and diversity of endophytic bacteria in the wild Amur grape. Thus, this study firstly described and analyzed the biodiversity of endophytic bacteria in wild grapevine *V. amurensis*.

## 1. Introduction

*Grapes* from the genus *Vitis* have been widely recognized as economically important fruit crops used for grape, wine, raisins, and juice production [1]. The Vitaceae family includes about 15 genera and ca. 900 species mostly in pantropical regions of Asia, Africa, Australia and the Pacific islands, with a few genera in temperate regions of the Northern Hemisphere [2]. It is well known that grape yield and fruit quality depend highly on external factors, such as the vineyard location, weather conditions, agricultural practices, and various biotic factors, such as microbial pathogens and endophytic microbiome [3].

Plant endophytes include bacteria, archaea, fungi, and protists that colonize the plant interior regardless of the outcome of the association [4]. Some endophytes are known to confer mutually beneficial effects to the host plant [3]. Some endophytes promote plant growth via nitrogen fixation, phytohormone production, or nutrient acquisition, and are also known to confer tolerance to abiotic and biotic stresses [5]. Endophytes possess considerable potential for application in agriculture as natural agents for biological control, plant growth promotion, crop yield improvement, and environmental stress control.

In the last decade, researchers have been actively studying the microbiome assemblage in grape species. Both general composition of endophytic communities and the endophyte biodiversity in healthy vs. diseased grapevine cultivars were studied to isolate microorganisms capable of grapevine pathogen biocontrol and fruit yield improvement.

The main body of research on the biodiversity of grapevine endophytic bacteria has been devoted to the *European* wine grape (*Vitis vinifera*) [6,7,8,9,10,11]. Most available studies show *Gammaproteobacteria* represent the dominant class of endophytic bacteria in *V. vinifera* [6,8]. It has been shown that the main bacteria included *Bacillus* spp., *Pseudomonas* spp., *Erwinia* spp., *Pantoea* spp., and *Curtobacterium* spp. In addition, there were studies on the endophytic bacterial communities of the grapevine varieties grown across the North American continent [12,13]. *Proteobacteria* were reported as the main representative of the bacterial community in American grape [12]. According to Deyett and Rolshausen [12], the core microbiome of grapevine sap from California was primarily composed of seven bacterial taxa (*Streptococcus*, *Micrococcus*, *Pseudomonas*, *Bacteroides*, *Massilia*, *Acinetobacter* and *Bacillus*) that were present throughout the growing season [13]. Moreover, Campisano et al. [14] conducted a comparative analysis of bacterial endophytes detected in wild and domesticated *European* grapevine *V. vinifera*. According to the study, wild grapevines were inhabited by a much more diverse endophytic bacterial community than domesticated counterparts. Thus, studying the bacterial microbiome of wild grapes represents an important area of research with a potential to detect new bacteriome variations that are not typical to cultivated varieties.

Currently, little is known about the microbiome of grapes growing in the Far East of Russia. The main representative of the Far Eastern wild grapes is the Amur grape *Vitis amurensis* Rupr. This species of grape exhibits a high resistance to low temperatures and microbial diseases, such as powdery mildew, grape white rot and anthracnose [15,16,17]. Furthermore, *V. amurensis* is used as rootstock to generate grape varieties with a high resistance to biotic and abiotic stress. Moreover, this species contains a high number of bioactive compounds (i.e., resveratrol and other stilbenes) with beneficial effects to human health [18]. It has been shown that stilbenes found in the stem of *V. amurensis* are capable of suppressing pathogenic bacteria such as *Pseudomonas aeruginosa*, *Xanthomonas axonopodis*, *Streptococcus mutans* and *Streptococcus sanguis* [19,20,21].

In addition, there are many examples where endophytic bacteria from wild relatives of crops, such as olive, rice, maize, barley and others, have been used to increase crop yields and resistance to environmental stresses. For example, bacterial endophytes from wild maize suppressed *Fusarium graminearum* and *Sclerotinia homoeocarpa* in cultivated maize and inhibited mycotoxin accumulation [22,23]. Moreover, endophytic bacteria from wild rice (*Oryza meridionalis*) bear considerable potential for promoting plant growth and degradation of phthalates [24,25].

Therefore, studying endophytic bacteriome of wild grape *V. amurensis* could contribute to the production of natural endophyte-containing agents that can be used to increase grapevine stress resistance and to improve the quality of grape-derived products. In this study, we aimed to analyze the biodiversity of endophytic bacteria in *V. amurensis* growing in natural population. The composition of bacterial endophytic community in *V. amurensis* was determined using bacterial sowing and NGS. This paper presents data on the biodiversity of bacterial endophytes in *V. amurensis* depending on the sample collection times, individual grape varieties, and different grapevine organs.

## 2. Results

### 2.1. The General Composition of Endophytic Bacterial Community in Different Organ of V. amurensis

**Figure 1:**
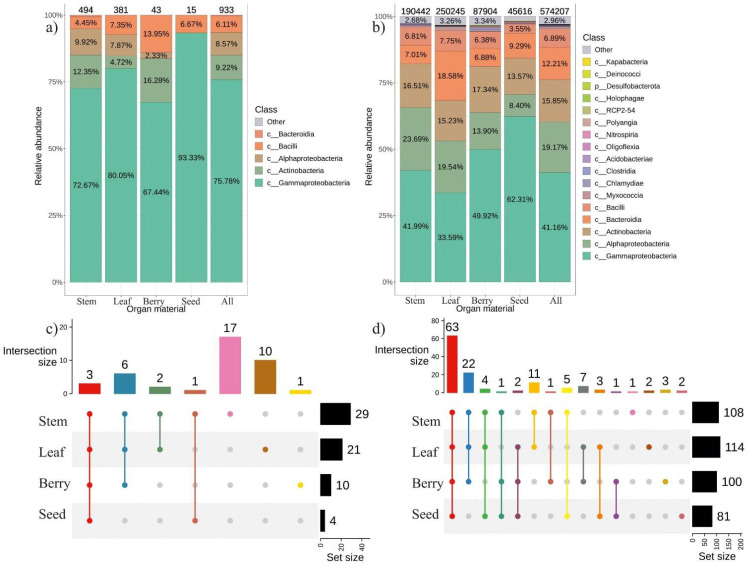
Comparative analysis of the endophytic community composition in different organs of wild grape *Vitis amurensis* obtained by the cultivation-dependent (bacteriological sowing (Sow)) and cultivation-independent approach (the next generation of sequencing (NGS)). The composition of endophytic bacteria of grape *V. amurensis* depends on the plant organ: (**a**) Class-level taxonomical bar plots for the bacterial community of bacteriological sow in berry, leaf, seed, stem and the sum of data for all organs; (**b**) Class-level taxonomical bar plots for the bacterial community as a result of the next generation of sequencing (NGS) in berry, leaf, seed, stem and the sum of data for all organs; (**c**) Genus-level UpSet diagrams depicting overlapping taxa of bacteriological sow in berry, leaf, seed, stem and the sum of data for all organs; (**d**) Genus-level UpSet diagrams depicting overlapping taxa of NGS in berry, leaf, seed and stem. Taxa were filtered based on relative abundance of >0.1% for each biocompartment. Filtered taxa in bar plot placed in “other” category and removed from UpSet diagram. Number of colonies (for sow) or amplicon sequence variants (ASVs) located above taxonomical bar plots.

We selected and identified the main representatives of the *V. amurensis* bacteriome. *V. amurensis* tissues were collected from two different plants in July and September for four years from 2018 to 2021. Then, a cultivation-dependent approach (bacterial seeding) was employed to analyze the endophytic bacteriome of *V. amurensis* using surface-sterilized plant tissues. We analyzed a total of 933 bacterial strains obtained as a result of the microbiological seeding procedure performed over the 4-year period. These strains were divided into four classes of bacteria. *Gammaproteobacteria* was the dominant class—76%. In addition, we detected *Actinobacteria*—9.2%, *Alphaproteobacteria*—8.6%, *Bacilli*—6.1%, and *Bacteroidia* less than 1% (Figure 1a).

Moreover, we used surface-sterilized tissues of the same two wild *V. amurensis* plants collected in July and September 2021 for NGS analysis of the endophytic bacterial community. We obtained 3,108,452 paired reads of *16s* rRNA gene. A total of 574,207 amplicon sequence variants (ASVs) were obtained after pre-processing for further analysis (Figure 1b). According to the analysis, 17 taxa were presented in different grapevine organs (stem, leaf, berry, seed) with the relative representation above 0.1%. Among the 17 taxa, ASVs belonging to 5 classes were the most represented: *Gammaproteobacteria*—40.3%, *Alphaproteobacteria*—18.7%, *Actinobacteria*—15.4%, *Bacteroidia*—12.2% and *Bacilli*—6.6% (Figure 1b). Thus, the metagenomic analysis generally confirmed the data of the cultivation-dependent approach. Notably, a total of 22 common genera of endophytic bacteria were detected by both cultivation-dependent and cultivation-independent approaches, while 18 unique genera were detected by the cultivation-dependent method and 59 unique genera–by NGS (Supporting Information S1).

**Figure 2:**
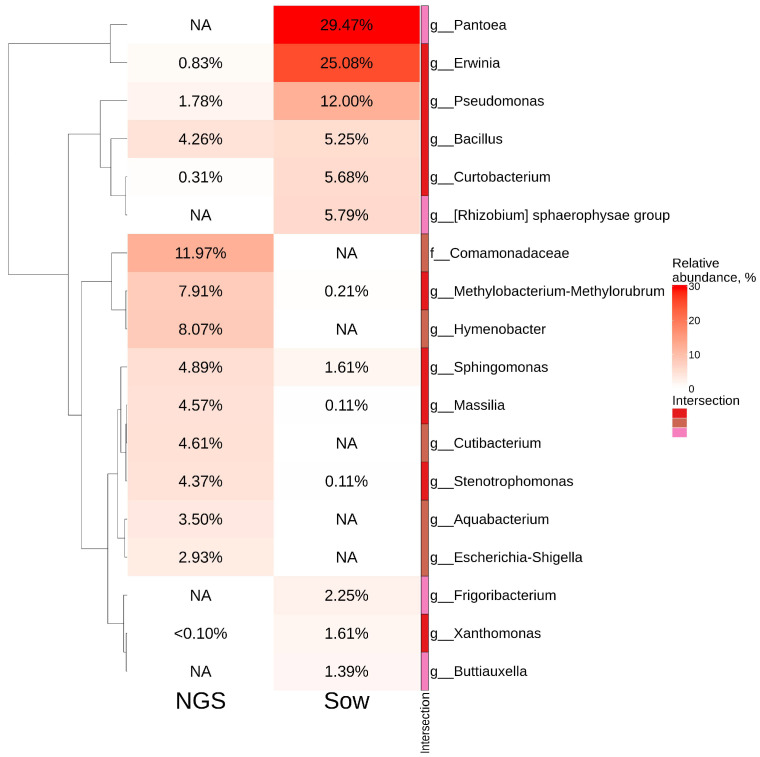
Genus-level relative abundance heat maps of significant taxa obtained through the cultivation-independent method (next generation of sequencing (NGS)) and cultivation-dependent method (bacteriological sowing (Sow)). The top 10 most abundant taxa from each factor are displayed. White squares (NA) represent absence of taxa.

According to the cultivation-dependent method, the predominant bacterial genera were *Pantoae*—29.5% and *Erwinia*—25%, while these bacterial genera were either not present or detected in trace amounts (less than 1%) in the metagenomics study (Figure 2). The top five taxa in NGS-analysis included *Comamonadaceae* (12%), *Methylobacterium* (8%), *Hymenobacter* (8%), *Sphingomonas* (5%) and *Cutibacretium* (5%). The *Comamonadaceae*, *Hymenobacter*, and *Cutibacterium* taxa accounted for 12%, 8%, and 4.6%, respectively, for all analyzed ASVs according to the cultivation-independent method, while they were not detected after bacterial seeding (Figure 2).

The analysis of endophytic bacteriome of the different *V. amurensis* organs revealed that the highest number of strains and obtained ASVs were detected in the leaves and stems, while the seeds showed much lower diversity of endophytic bacteria (Figure 1c,d). The *Gammaproteobacteria* class was dominant in the seeds, while the *Alphaproteobacteria* and *Bacilli* classes were less represented. The leaves of *V. amurensis* were predominantly colonized by the *Bacteroidia* and *Myxococcia* compared to other plant organs. ASVs were represented by 128 taxa in the samples of plant organs. Among them, 63 taxa were found in all analyzed grapevine organs (Figure 1d and Supporting Information S2). The beta diversity data showed diffuse clustering and no significant difference between organ samples performed by PERMANOVA test (R^2^: 13.6%, *p* = 0.521) (Supporting Information S3).

**Figure 3:**
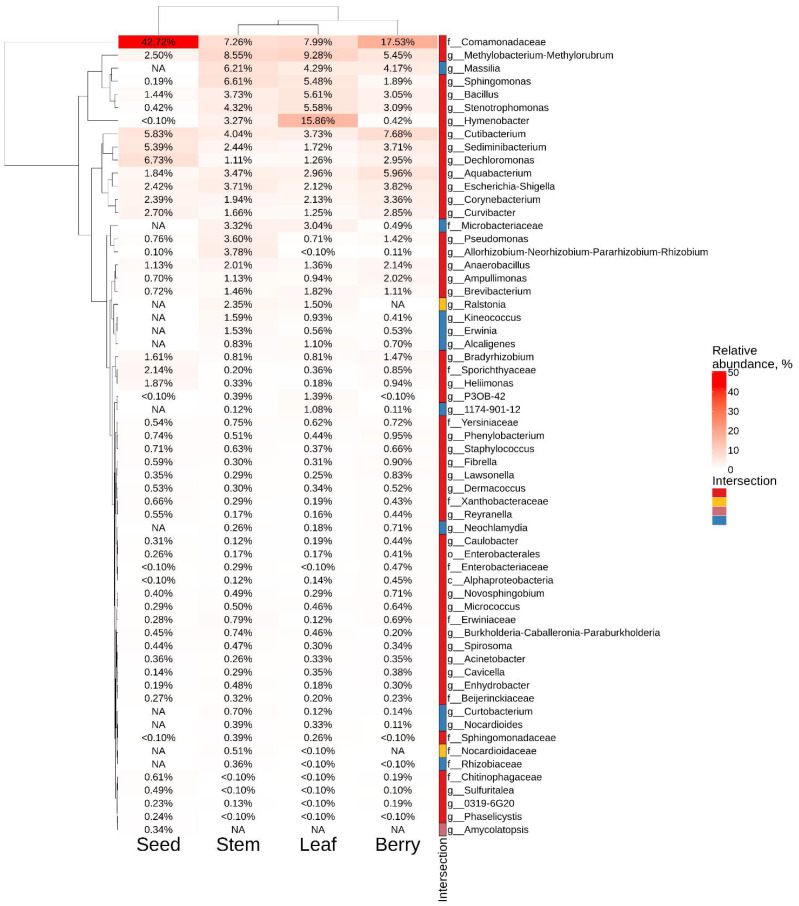
Genus-level relative endophytic bacteria abundance heat maps of significant taxa the next generation of sequencing (NGS) in different grape organs (stem, leaf, berry and seed). The top 40 most abundant taxa from each factor are displayed. White squares (NA) represent absence of taxa. The intersection selection is made based on the Figure 1d.

The most common taxa for the leaves of *V. amurensis* were *Hymenobacter* (16%), *Methylobacterium*-*Methylorubrum* (9%), and *Comamonadaceae* (8%), while the most common taxa for the stem were represented by *Sphingomonas* (6.6%), *Methylobacterium*-*Methylorubrum* (8.5%), and *Comamonadaceae* (7.2%) (Figure 3). The largest number of ASVs in grape berries and seeds belonged to the taxa *Comamonadaceae* (17.5% and 43%, respectively). Moreover, genera *Cutibacterium* (5.8%) and *Dechloromonas* (6.7%) were most often found in grape seeds (Figure 3).

### 2.2. Differences in the Composition of Endophytic Bacterial Community in V. amurensis Depending on the Year of Tissue Collection

**Figure 4:**
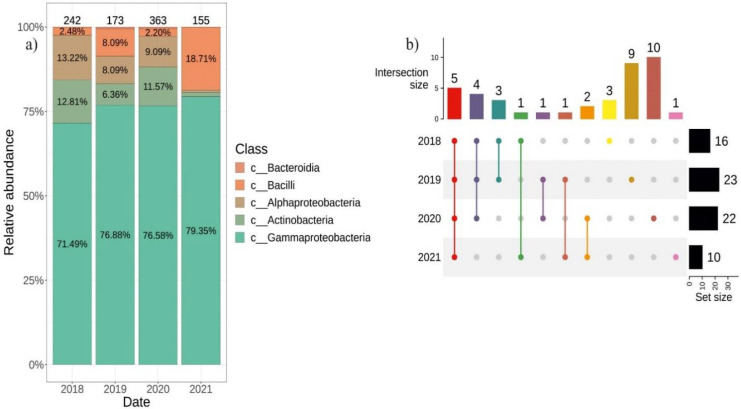
Composition of endophytic bacterial community in wild grape *Vitis amurensis* depending on the year of material collection. (**a**) Class-level taxonomical bar plots for the bacterial community composition in 2018–2021; (**b**) Genus-level UpSet diagrams depicting overlapping taxa of bacteriological sow and next-generation sequencing. Taxa were filtered based on relative abundance of >0.1% for each biocompartment. Number of colonies located above taxonomical bar plots.

We used the cultivation-dependent approach to isolate individual endophytic bacteria from the tissues of two *V. amurensis* plants collected every year from 2018 to 2021. In 2018, we isolated and identified 242 strains of endophytic bacteria, in 2019–173 strains, in 2020–363 strains and in 2021–155 strains (Figure 4a). Most strains of endophytic bacteria (71.5–79.4%) collected each year belonged to *Gammaproteobacteria*. The distribution among other classes of endophytic bacteria varied depending on the year of sampling. In 2018 and 2020, the percentage ratio of the endophytic bacteria classes were similar. In 2020, *Bacteroidia* class were detected (0.55%), while in 2018 this class was not present. The incidence of the *Actinobacteria* class in 2019 decreased by half and reached 6% compared to 2018 and 2020, respectively, and dropped to less than 1.3% in 2021. The incidence of *Bacilli* was 2% in 2018 and 2020, while it increased to 8% and 19% in 2019 and 2021, respectively (Figure 4a). The generic biodiversity was the richest in 2019 and 2020 (23 and 22 genera), and there were 9 and 10 unique genera for 2019 and 2020, respectively (Figure 4b). Common genera that were detected every year of the bacterial seeding were *Bacillus*, *Erwinia*, *Pantoea*, *Pseudomonas* and *Sphingomonas* (Supporting Information S4).

### 2.3. Seasonal Variations in the Composition of Endophytic Bacteriome in V. amurensis

**Figure 5:**
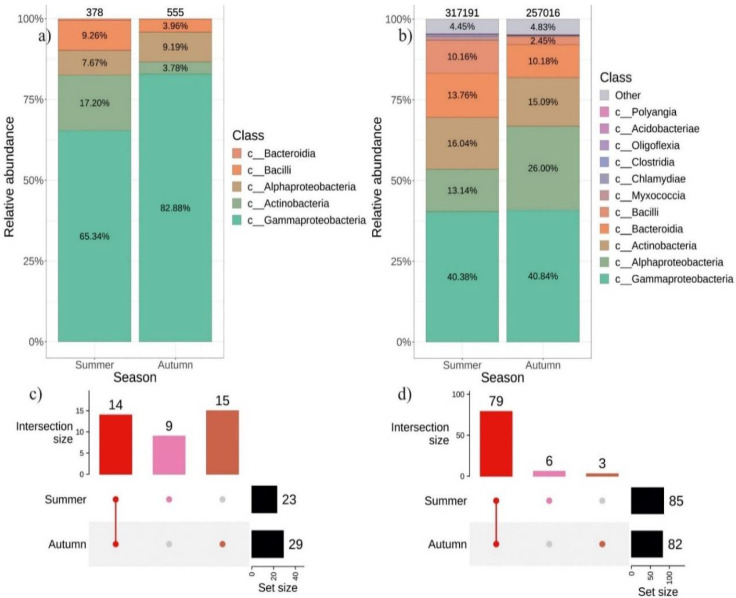
Composition of endophytic bacterial community in wild grape *Vitis amurensis* depends on the season of material collection: (**a**) Class-level taxonomical bar plots for the bacterial community of cultivation-dependent approach in summer and autumn; (**b**) Class-level taxonomical bar plots for the bacterial community as a result of cultivation-independent approach (next generation of sequencing (NGS)) in summer and autumn; (**c**) Genus-level UpSet diagrams depicting overlapping taxa of bacteriological sow in summer and fall; (**d**) Genus-level UpSet diagrams depicting overlapping taxa of NGS in autumn and summer. Taxa were filtered based on relative abundance of >0.1% for each biocompartment. Filtered taxa in bar plots placed in “other” category and removed from UpSet diagram. Number of colonies or amplicon sequence variants (ASVs) located above taxonomical bar plots.

We also analyzed the biodiversity profile of endophytic bacteria in *V. amurensis* depending on the collection season. The leaves and stems of *V. amurensis* were collected in the first half of July and in the second half of September. The cultivation-dependent approach resulted in a greater number of strains sown in autumn (555) than in summer–(378) (Figure 5a). In total, 14 genera of bacteria were common for summer and autumn, while 9 genera were unique for summer and 15 for autumn (Figure 5c and Supporting Information S5).

A total of 257,016 ASVs were obtained in the autumn of 2021 and 317,191 ASVs in the summer of 2021 using the cultivation-independent approach. The percentage of *Alphaproteobacteria* increased two-fold in the autumn, while the percentage of *Bacilli* decreased four-fold (Figure 5b). A total of 6 genera were identified as unique for the summer, and 3 genera were unique for the autumn (Figure 5d and Supporting Information S6). PERMANOVA test showed no significant difference between summer and autumn samples (R^2^: 8.8%, *p* = 0.068) (Supporting Information S3).

### 2.4. The Composition of Endophytic Bacterial Community in Different Representatives of V. amurensis Grapevine

**Figure 6:**
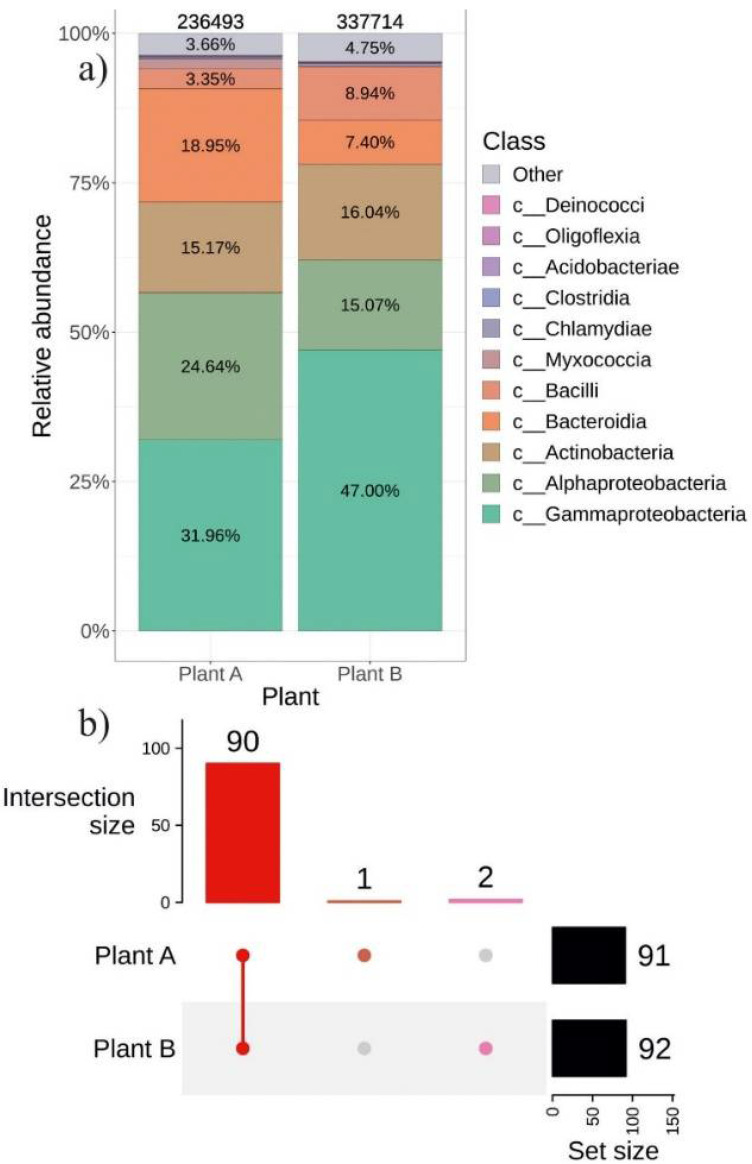
Composition of endophytic bacterial community in two different *Vitis amurensis* plants: (**a**) Class-level taxonomical bar plots for the bacterial community as a result cultivation-independent approach (the next generation of sequencing (NGS)) in Plant A and Plant B; (**b**) Genus-level UpSet diagrams depicting overlapping taxa of NGS in Plant A and Plant B. Taxa were filtered based on relative abundance of >0.1% for each biocompartment. Filtered taxa in bar plots placed in “other” category and removed from UpSet diagram. Number of amplicon sequence variants (ASVs) located above taxonomical bar plots.

**Figure 7:**
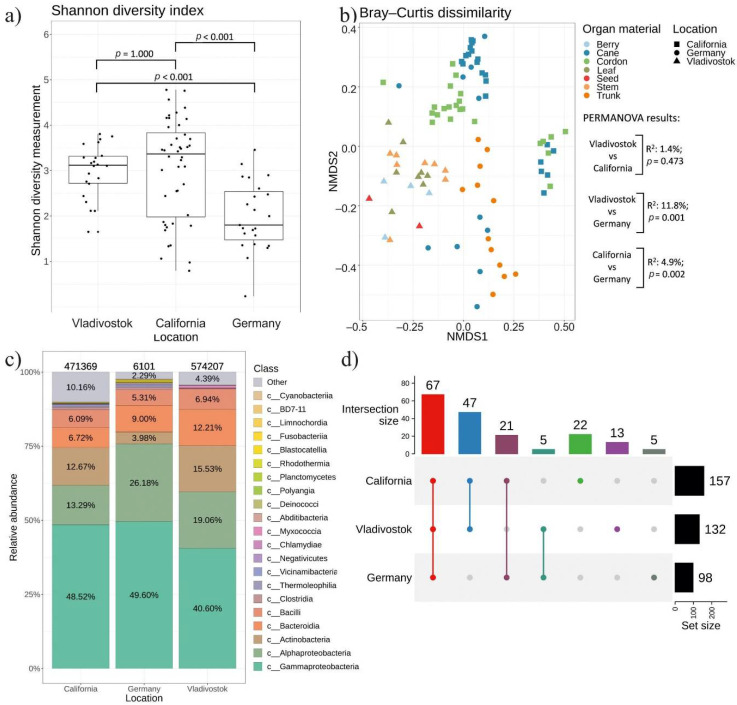
A comparison of endophytic bacterial communities in cultivated *Vitis vinifera* from USA (California) and Germany with the endophytic community in *Vitis amurensis* from the Russian Far East (Vladivostok) (**a**) Shannon’s alpha diversity boxplot; (**b**) Bray–Curtis beta diversity NMDS plot; (**c**) Class-level taxonomical bar plots for the bacterial community composition *V. vinifera* from USA (California), Germany, and *V. amurensis* from Russia (Vladivostok); (**d**) Genus-level UpSet diagrams depicting overlapping taxa of bacteriological sow and next-generation sequencing. Taxa were filtered based on relative abundance of >0.1% for each biocompartment. Filtered taxa in bar plot placed in “other” category and removed from UpSet diagram. Number of amplicon sequence variants (ASVs) located above taxonomical bar plots.

In addition, we compared the percentage of the community of endophytic bacteria in individual plants of *V. amurensis*. The two representative *V. amurensis* plants differed only in their place of growth, while their approximate ages were the same. We detected no significant differences in beta diversity between samples collected from plant A and plant B (R^2^: 8.9%, *p* = 0.064) (Supporting Information S3). The incidence of *Bacteroidia* was 2-fold higher in plant A than in plant B (Figure 6a). There were only two unique taxa in plant B belonging to *Gammaproteobacteria* and *Intrasporangiaceae* and one unique genus *Frondihabitans* in plant A (Figure 7d and Supporting Information S7).

### 2.5. Comparative Analysis of Endophytic Bacteria Biodiversity in V. amurensis and V. vinifera

We conducted a comparative analysis of the bacteriomes of *V. amurensis* obtained in this study with the previously studied bacteriomes of *V. vinifera* grapevines growing in Germany [26] and California, USA [12] (Supporting Information S8). We analyzed the amplicon data of each sample site with respect to the location. The results for alpha and beta diversity analysis are shown in Figure 7a,b, respectively. The grape samples collected in Russia (Vladivostok) and USA (California) were similar in alpha diversity. The Germany samples exhibit reduced alpha diversity compared to Russia (Vladivostok) (*p* < 0.001) and to USA (California) (*p* < 0.001). (Figure 7a and Supporting Information S9). The beta diversity results are presented in the nonmetric multidimensional scaling (NMDS) ordination (Figure 7b). NMDS ordination showed that samples from Russia (Vladivostok), USA (California) and Germany were located in separate clusters (Figure 7b). The PERMANOVA test demonstrated that the samples collected in Vladivostok and California were more similar (R^2^: 1,4%, *p* = 0.473) based on beta diversity than samples from California and Germany (R^2^: 4,9%, *p* = 0.002), while the samples from Russia and Germany were most diverse (R^2^: 11,8%, *p* = 0.001) (Supporting Information S10). These results were also supplemented by UpSet intersection diagrams. Samples collected in Vladivostok and California displayed 47 intersection genera, California and Germany had 21, and Vladivostok and Germany had only 5 (Figure 7d and Supporting Information S11).

**Figure 8:**
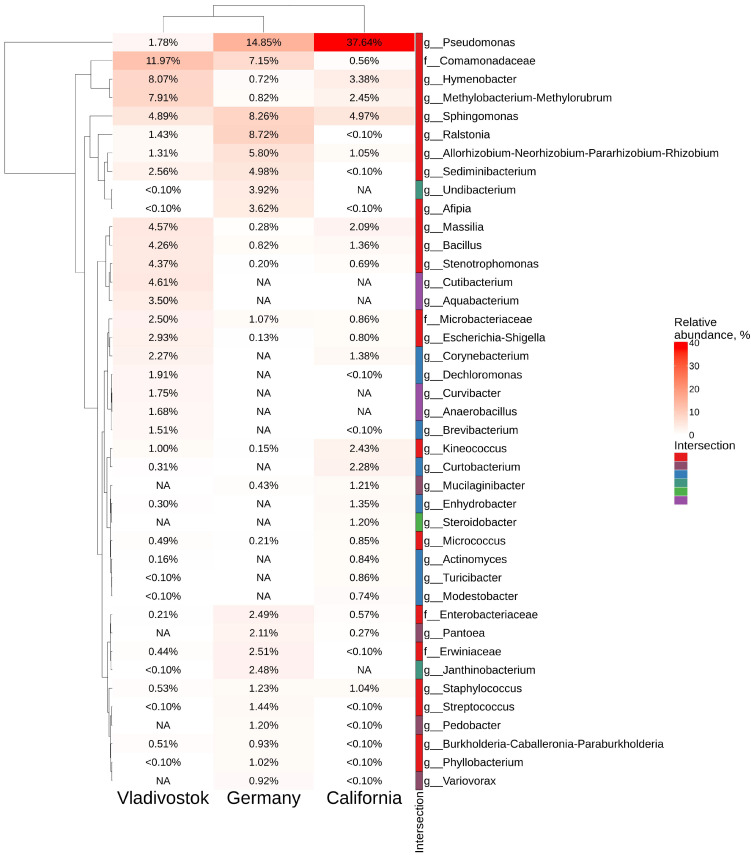
Genus-level relative endophytic bacteria abundance heat maps of significant taxa of *Vitis vinifera* from USA (California), Germany, and *Vitis amurensis* from Russian Far East (Vladivostok). The top 20 most abundant taxa from each factor are displayed. White squares (NA) represent absence of taxa. The intersection selection is made based on the Figure 7d.

According to the analysis, the grape bacteriome is represented by 23 main classes of bacteria (Figure 7c). The classes *Gammaproteobacteria*, *Alphaproteobacteria*, *Actinobacteria*, *Bacteroidia* and *Bacilli* dominated over other classes in all bacteriomes included in the analysis. *Gammaproteobacteria* was the dominant class in all grapevine samples (40–50%), with *Pseudomonas* as the most represented genus in California and Germany samples (Figure 8). The most abundant taxa in the samples collected in Russia (Vladivostok) was *Comamonadaceae* (*Betaproteobacteria*). There were 67 genera common to all regions of the material collection. The analysis also revealed 22 unique genera for USA (California), 13—for Russia (Vladivostok) and 5—for Germany (Figure 7d and Supporting Information S11). For the wild grape *V. amurensis*, the unique genera were *Alcaligenes*, *Ampullimonas*, *Anaerobacillus*, *Aquabacterium*, *Curvibacter*, *Cutibacterium*, *Dermacoccus*, *Erwinia*, *Fibrella*, *Frondihabitans*, *Heliimonas*, *Neochlamydia* and *Nitrospirillum* (Figure 8 and Supporting Information S11).

## 3. Discussion

The microbiota of grapes are highly variable, mostly due to the influence of external factors, such as environmental cues, geographical location, or individual characteristics of grape varieties [8]. This study focused on the bacterial endophytes from wild *V. amurensis* growing in natural conditions. A comparative analysis of findings from the cultivation-dependent (bacteriological seeding in 2018–2021) and cultivation-independent (NGS) approaches revealed that *Gammaproteobacteria*, *Alphaproteobacteria*, *Actinobacteria*, *Bacteroidia* and *Bacilli* were the dominant classes in the endophytic bacteriome of the wild grape. However, the ratio between the genera of endophytic bacteria considerably varied according to the two methods. This can be explained by the fact that we sowed bacterial strains over the 4-year period, while NGS was performed only for probes collected in the summer and autumn of 2021. Weather conditions varied considerably each year. In addition, the lower sensitivity of the culture-dependent approach contributed to the difference between the results. In our opinion, these two conditions led to the observed differences in the percentages of endophytic bacterial genera. Therefore, it is necessary to apply both a cultivation-dependent and a cultivation-independent approach over a time period of several years in order to obtain a more comprehensive picture of endophytic bacteria assemblage in the same plant.

The cultivation-dependent method demonstrated that 10 bacterial genera prevailed in the *V. amurensis* endophytic community (*Pantoea*, *Erwinia*, *Pseudomonas*, *Bacillus*, *Curtobacterium*, *Rhizobium* (sphaerophysae group), *Frigoribacterium*, *Sphingomonas*, *Xantomonas*, and *Buttiauxella*) (Figure 2). The data obtained on the main genera of endophytic bacteria present in *V. amurensis* were similar to the previously published data on bacterial community composition in *V. vinifera* from Australia and Italy [6,7,8,9,27].

While studying the endophytic bacteria distribution over several years (2018, 2019, 2020 and 2021), we discovered an interesting relationship between the weather conditions and bacterial biodiversity: a minimal number of endophytic bacteria (only 155 strains) were detected under hot and dry weather conditions in the Primorsky Territory of Russia (2021 year) (Figure 4a and Table 1). On the contrary, we isolated the maximum number of endophytic bacteria (363 strains) under cold and damp conditions in 2020 using the same methods (Figure 4a and Table 1). Thus, an average temperature of 15 °C and a large amount of precipitation contribute to both quantitative and qualitative biodiversity of endophytic bacteria of *V. amurensis*.

Similar to the results obtained by Baldan et al. [11], the bacterial composition changed depending on the season of sampling. We showed that the composition of bacterial endophytes was richer in autumn than in summer. This was probably due to the gradual colonization of endophytic bacteria during growth and development of the aboveground grapevine organs, e.g., leaves. In addition, there were some unique genera of bacteria in wild grape *V. amurensis* either in the summer or in the autumn periods. This indicates a special influence of various environmental physical parameters (air temperature, precipitation, illumination, etc.), on the percentage ratio between different classes of bacteria present in the intercellular space of grapevine tissues.

The analysis of the bacteriological community in different organs of *V. amurensis* showed that most endophytic bacteria inhabited stems and leaves. This conclusion confirmed previously known information that the endophytes of grapes travel from the root system through conducting vessels into the stem and then move into the leaves [12]. Moreover, we noted that number of endophytic bacterial strains and the amount of ASVs were significantly lower in summer leaves than in autumn ones. The data indicate that the bacteria had not arrived in time to settle in the leaves at the beginning of the summer season compared to the autumn. A similar situation was also observed in the case of berries and seeds: the number of ASVs detected and the number of isolated strains of endophytic bacteria were 3–12 and 2.2–8.9 times lower in the berries than in the stems and leaves, respectively (Figure 1). This effect was most likely due to the fact that the berries developed later than the leaves and contained more phenolic compounds, which prevented active endophyte accumulation.

The lowest biodiversity of endophytic bacteria was found in the grape seeds, which can be explained by the better protection of seeds by the berry pulp. Interestingly, the metagenomic analysis revealed two genera of endophytic bacteria unique to the grape seeds, i.e., the genera *Amycolatopsis* and *Pseudoalteromonas* (Figure 1 and Supporting Information S2). It is known that bacteria of the genus *Amycolatopsis* are able to produce antibiotics [28], while bacteria of the genus *Pseudoalteromonas* are usually found in marine eukaryotes and are capable of producing bioactive compounds [29]. Therefore, it is possible that symbiosis of the seed-associated bacteria with the plant could contribute to seed protection.

Next, we compared the data on endophytic microbial assemblage in *V. amurensis* with the previously studied endophytic bacteria communities in grapevines from other regions of the world, including Germany [26] and USA (California) [12]. Nonmetric multidimensional scaling showed that samples from Russia (Vladivostok), USA (California), and Germany were located in separate clusters. The bacteriome of grapes was represented by 23 main classes of bacteria. The prevalent classes were *Gammaproteobacteria*, *Alphaproteobacteria*, *Actinobacteria*, *Bacteroidia* and *Bacilli* for all grapes. *Gammaproteobacteria* was the dominant class in all samples (40–50%), with *Pseudomonas* as the most represented genus in USA and Germany, and *Comamonadaceae* (*Betaproteobacteria*)–in Russia. There were 46 genera common to all regions of material collection. We also detected a number of unique genera for USA (14 genera), Russia (11 genera), and Germany (4 genera). The differences between the endophytic bacteriome compositions in the grapevines show that the place of growth also affects the percentage ratio between classes of endophytic bacteria. However, this issue needs further investigation with additional RNA-seq libraries from other cultivars. In order to present a more complete picture of grapevine microbiota, it is necessary to expand the studies on biodiversity of bacterial endophytes to a higher number of cultivars and individuals from the wild and cultivated *Vitis* species growing globally.

The data obtained in this investigation are interesting for practical application in agriculture. Knowledge about the biological diversity of endophytic bacteria in wild grapes, including *V. amurensis,* could prove useful for the development of new approaches to increase the stress resistance of cultivated grapes, yield, and fruit quality. For example, it has been shown that some endophytic bacterial isolates from domesticated and wild *V. vinifera* grapevines were highly active against *Botrytis cinerea, Neofusicoccum parvum*, *Botryosphaeria dothidea*, *Botryosphaeria obtuse*, *Pochonia chlamydospora*, *Plasmopara viticola,* and *Rhizobium vitis* in vitro [9,14,30]. *Bacillus* and *Pantoea* exhibited the most prominent antimicrobial activities. Moreover, bacterial endophyte *Bacillus licheniformis* isolated from *V. vinifera* has been shown to stimulate the production of secondary metabolites, such as monoterpenes, exhibiting antioxidant activity, and sesquiterpenes, exhibiting antibacterial effects [31]. Moreover, *B. licheniformis* produced carotenoids, which can act as antioxidants useful for plant stress protection [32]. It has been shown that bacterial endophytes *B. licheniformis* and *Pseudomonas fluorescens* can modulate ABA metabolism in inoculated grape plants, which gives them an advantage over uninfected plants in drought conditions [31,33].

Grape endophytic bacteria have previously been shown to favorably affect the quality of grape fruit. For example, grapevine inoculation with endophytic *Acinetobacter lwoffii*, *Bacillus subtilis*, and *Pseudomonas fluorescens* was effective against *Botrytis cinerea* and led to the accumulation of host-synthesized phytoalexins, especially *trans*-resveratrol (3,5,4′-tryhydroxystilbene) and its oligomer, *trans*-ε-viniferin, which could contribute to the grape fruit metabolite composition [34]. Interestingly, native endophytes and products based on endophytes from the wild grape *V. amurensis* can stimulate stilbene production in grape cell suspensions, which could further contribute to the development of a new stimulators of stilbene biosynthesis in grapevine or grape cell cultures [35,36].

Taken together, these obtained data can be employed to create endophyte-based preparations for plant pathogen protection. Thus, future studies on the biochemical properties (e.g., the ability to secrete phytohormones or biologically active substances) or biological functions (e.g., plant disease protection) of isolated endophytic bacteria can greatly contribute to crop protection and plant functional studies.

## 4. Materials and Methods

### 4.1. Plant Material

For both cultivation-dependent and cultivation-independent approaches, we used tissues of two healthy 10–15-year-old vines of *V. amurensis* located at a distance of 1 km from each other in a nonprotected natural population near Vladivostok, Russia (the southern Primorsky Territory of the Russian Far East, longitude 43.2242327 and latitude 131.99112300). Shoots, leaves (young stems 7–8 cm long with three healthy leaves), berries (green and mature), and seeds were collected at 10–11 a.m. on low-cloud days without precipitation, the air temperature was 17–20 °C. The values of the average temperatures and precipitation in 2018–2021 in Vladivostok (Primorsky Territory of Russia) are shown in the Table 1. Each plant material specimen was delivered to the laboratory within 30 min.

For the cultivation-dependent approach (bacterial sowing), plant materials were selected in July and September from 2018 to 2021. Two biological replicates (two individual vines) were collected in July, and two biological replicates–in September. Thus, there were 4 biological replicates per year. In total, 16 biological replicates were collected and analyzed by the cultivation-dependent approach from 2018 to 2021. For the cultivation-independent approach (NGS) we used grapevine material collected in July and September of 2021 (a total of 4 biological replicates) and applied 2 technical replications per biological replicate.

### 4.2. Isolation and Identification of the Endophytic Bacteria

The grapevine tissues (1.5 g) were washed under running water with soap and washed sequentially under sterile conditions in 75% ethanol for 2 min, 10% hydrogen peroxide for 1 min, and five times in sterile water. To check the efficacy of this method of surface sterilization, 100 µL of the last wash water was incubated on the R2A medium [37]. No microorganism growth was observed 3 days after the last portion of washing water had been plated in the Petri plates containing the growth media. This validated the quality of the performed superficial sterilization of the grape tissues.

The surface-sterilized tissue of *V. amurensis* was ground to a homogeneous mass in a sterile mortar; the resulting juice was squeezed, and a 100 μL aliquot was transferred to R2A media in Petri plates. After 3 days, the grown bacterial colonies were sampled and carefully transferred to a new sterile Petri plate for repeated cultivation. We isolated almost all the seeded colonies into separate strains, a total of 933 separate strains of endophytic bacteria were obtained over 4 years of biological sowing.

DNA of the 933 bacteria strains was isolated by the hexadecyltrimethylammonium bromide (CTAB) method with modifications [38]. Bacterial *16S rRNA* gene sequences were amplified using universal bacterial primers for the amplification of approximately 1500 bp *16S* PCR products (8F, 5′AGA GTT TGA TCM TGG CTC AG and 1522R, 5′AAG GAG GTG ATC CAR CCG CA) [39]. PCR products were sequenced using an ABI 3130 Genetic Analyzer (Applied Biosystems, Foster City, CA, USA) according to the manufacturer’s instructions as described [35,36]. The Basic Local Alignment Search Tool (BLAST) program was used for sequence analysis. Multiple sequence alignments were performed using the Clustal X program [40]. A sequence identity of ≥99% is considered as a sufficient threshold value for taxonomic identification bacteria genus.

### 4.3. DNA Extraction, PCR Condition, Library Preparation, and Sequencing

DNA for NGS was isolated using two approaches. The first approach used the method employed in our laboratory [38] with small modifications. Namely, 50 mg of *V. amurensis* tissue was taken 800 µL CTAB-buffer with 2× content of NaCl (100 mM Tris pH 7.5, 1.4 M NaCl, 40 mM EDTA pH 7.5, 1% CTAB) was added, incubated for 1 h 60 °C in a Gnom thermostat (DNA-technology, Moscow, Russia). Then 300 µL of chloroform was added, gently mixed and centrifuged for 10 min at 4 °C and 13,200 rpm (Ependorf, Germany). Further, a supernatant (420 µL) was selected in separate test tubes with 950 µL of 96% ethanol and frozen at −20 °C overnight. Then, the DNA in ethanol was centrifuged for 10 min at 4 °C and 13,200 rpm, and then the supernatant was removed. The precipitate was dried until the ethanol completely evaporated at room temperature (30 min). The precipitate was dissolved in 100 µL of distilled water and then purified on Plasmid DNA purification columns Plasmid MiniPrep kit per manufacturer’s protocol (Evrogen, Moscow, Russia). As a result, the DNAs were eluted in 50 µL of elution buffer.

According to the second approach, the DNA was extracted from 30 mg of *V. amurensis* tissue using the ZymoBIOMICS DNA miniprep kit per manufacturer’s protocol (Zymo Research, Irvine, CA, USA). DNA was assessed for quality and quantity using the NanoPhotometer P300 (IMPLEN, Munich, Germany).

The DNA samples were sent to Evrogene (Moscow, Russia) for high-throughput sequencing using Illumina technology. The libraries were prepared for sequencing according to the protocol described in the manual “16S Meta-genomic Sequencing Library Preparation” (Part #15,044,223 Rev. B; Illumina, San Diego, CA, USA). Bacterial *16S* rRNA regions were amplified from all samples using primers 515F (5′GGTAATACGKAGGKKGCDAGC) and 806R (5′RTGGACTACCAGGGTATCTAA) modified for *Vitis* sp. plants. After obtaining the amplicons, the libraries were purified and mixed equimolarly using the SequalPrep™ Normalization Plate Kit (Cat #A10510-01, ThermoFisher, Waltham, MA, USA). Quality control of the obtained library pools was performed using Fragment Analyzer and quantitative analysis was performed using qPCR. The library pool was sequenced on Illumina MiSeq (2 × 250 paired end) using MiSeq Reagent Kit v2 (500 cycles). The FASTQ files were obtained using bcl2fastq v 2.17.1.14 Conversion Software (Illumina, San Diego, CA, USA). The phage PhiX library was used to control sequencing parameters. Most of the reads pertaining to phage DNA were removed during demultiplexing.

Bacterial sequences were deposited in NCBI under the accession number PRJNA813962 and in database of laboratory Biotechnology, Federal Scientific Center of the East Asia Terrestrial Biodiversity, Far Eastern Branch of the Russian Academy of Sciences, Russia (https://biosoil.ru/downloads/biotech/Vitis%20metagenom/2021-09=Vitis_amurensis_endophytes_16s), (accessed on 14 March 2022).

### 4.4. Computational Analysis

For the comparative analysis of endophytic bacteriomes from *V. amurensis* and *V. vinifera*, we selected two studies from California (cv. ‘Syrah’) and Germany (cv. ‘Cabernet Dorsa’) where the biodiversity of endophytic bacteria was evaluated [12,26]. The main criteria for the selection of metagenomic studies were the presence of a bacterial film elimination step in the “Materials and Methods” section of research, different geographical location and the same *16s* rRNA sequencing region (515F-806R). From the selected studies, we selected samples belonging to the above-ground parts of the plant and not subjected to any treatments or infections. The samples used in the comparative analysis are presented in Supporting Information S8. Raw readings of all samples were subjected to further bioinformatic analysis.

NGS reads were preprocessed using QIIME 2 [41] and DADA2 programs [42]. The primers, remaining PhiX reads, and chimeric sequences were removed, and paired-end reads were merged and sorted. The authors of the DADA2 algorithm refer to the sequences as amplicon sequence variants (ASVs). This algorithm has the capacity to resolve sequences differences to a single nucleotide, allowing for more robust identification. Taxonomic identification of ASVs was performed using the QIIME 2 Scikit-learn algorithm [43] using the Silva 138 pre-trained classifier (99% OTUs from 515F/806R region of sequences) [44].

The obtained data were processed using the R language. The phyloseq library [45] and tidyverse package [46] were used in pre-filtering and data preparation. Taxa for bar plot, heatmap and UpSet diagram visualizations were filtered based on relative abundance of >0.1% for each biocompartment. We merged the taxonomic ranks in bar plots that were relative abundant <0.1% in each factor to one group called “other”. Shannon alpha diversity and Bray–Curtis beta diversity data were obtained using the Vegan package [47]. Bray–Curtis dissimilarity data were transformed to even sampling depth and converted to nonmetric multidimensional scaling (NMDS). A pairwise Wilcoxon rank sum test with Bonferroni correction was performed to analyze the alpha diversity data between groups. Statistical validation of beta diversity data was performed using the PERMANOVA test with 999 permutations [47]. The ggplot2 [46] and ComplexHeatmap [48] R libraries were used in the graphical representation of the results.

## 5. Conclusions

In this study, we firstly described the biodiversity of endophytic bacteria in wild-growing grapevine *V. amurensis*. According to both the cultivation-dependent and cultivation-independent approaches, *Gammaproteobacteria*, *Actinobacteria*, *Alphaproteobacteria*, *Bacilli* and *Bacteroidia* represented the dominant classes of bacteria. The data indicated that the weather conditions significantly affected the number and biodiversity of endophytic bacteria in *V. amurensis*. Namely, lower temperatures (15–16 °C) and increased precipitation (140–280 mm in month) favored both the quantitative and qualitative diversity of endophytic bacteria inhabiting the intercellular space of *V. amurensis* tissues. The obtained data also show that the composition of bacterial endophytes was richer in autumn than in summer. Among the aboveground organs of grapes, the stems and leaves were the leaders in quantitative composition and biodiversity of endophytic community. In addition, this study showed that the community of endophytic bacteria of *V. amurensis* was closer to the endophytic community of *V. vinifera* growing in Germany. Thus, this paper presented and discussed various factors that affect the biodiversity of endophytic bacteria in wild grapevine *V. amurensis*. Considering that a high number of factors affects the biodiversity of endophytic bacteria in *V. amurensis*, this study should be expanded to include more individuals and geographic areas where this grapevine species is present. The knowledge about the biological diversity of endophytic bacteria in wild grapes *V. amurensis* will development of new approaches to increase grapevine stress resistance as well as the yield and quality of fruits.

## Figures and Tables

**Table 1:** The values of the average temperature and amount of precipitation from 2018–2021 in Vladivostok, Primorsky Territory of Russia.

Summer	Average t, °C	Precipitation, mm	Autumn	Average t, °C	Precipitation, mm
The norm	18.1	159	The norm	16	103
July 2018	18.5	124	September 2018	16.4	163
July 2019	17.1	131	September 2019	17.3	44
July 2020	14.7	281	September 2020	16.3	138
July 2021	21.3	24	September 2021	17.7	120

http://www.pogodaiklimat.ru/ (accessed on 20 January 2022)

## Data Availability

The data presented in this study are available within the article and supplementary material.

## Supplementary Materials

The following are available online at https://www.mdpi.com/article/10.3390/plants11091128/s1, Supporting Information S1. Intersections in our data (Data type); Supporting Information S2. Intersections in our metagenome data (Organ material)—Figure 1d; Supporting Information S3. NMDS ordination plots and PERMANOVA results in our data; Supporting Information S4. Intersections in data of cultivate-dependent method (Date)—Figure 4b; Supporting Information S5. Intersections in data of cultivate-dependent method (Season)—Figure 5c; Supporting Information S6. Intersections in our metagenome data (Season)—Figure 5d; Supporting Information S7. Intersections in our metagenome data (Plants)—Figure 6b; Supporting Information S8. Samples used in comparative analysis of the grape bacteriomes; Supporting Information S9. Alpha diversity results; Supporting Information S10. PERMANOVA results in comparative analysis of the bacterial biodiversity between grapes; Supporting Information S11; Intersections between *Vitis* metagenome data—Figure 7d.
